# Targeting TRPV1-mediated autophagy attenuates nitrogen mustard-induced dermal toxicity

**DOI:** 10.1038/s41392-020-00389-z

**Published:** 2021-01-25

**Authors:** Mingliang Chen, Xunhu Dong, Haoyue Deng, Feng Ye, Yuanpeng Zhao, Jin Cheng, Guorong Dan, Jiqing Zhao, Yan Sai, Xiuwu Bian, Zhongmin Zou

**Affiliations:** 1 Department of Chemical Defense Medicine, School of Military Preventive Medicine, Third Military Medical University (Army Medical University), 30 Gaotanyan Street, Shapingba District, Chongqing, 400038 China; 2 Institute of Pathology and Southwest Cancer Centre, Southwest Hospital, Third Military Medical University (Army Medical University), Chongqing, 400038 China; 3 Institute of Toxicology, School of Military Preventive Medicine, Third Military Medical University (Army Medical University), 30 Gaotanyan Street, Shapingba District, Chongqing, 400038 China; 4 State Key Laboratory of Trauma, Burns and Combined Injury, Second Department of Research Institute of Surgery, Daping Hospital, Third Military Medical University (Army Medical University), Chongqing, 400042 China

**Keywords:** Experimental models of disease, Cell biology

## Abstract

Nitrogen mustard (NM) causes severe vesicating skin injury, which lacks effective targeted therapies. The major limitation is that the specific mechanism of NM-induced skin injury is not well understood. Recently, autophagy has been found to play important roles in physical and chemical exposure-caused cutaneous injuries. However, whether autophagy contributes to NM-induced dermal toxicity is unclear. Herein, we initially confirmed that NM dose-dependently caused cell death and induced autophagy in keratinocytes. Suppression of autophagy by 3-methyladenine, chloroquine, and bafilomycin A1 or *ATG5* siRNA attenuated NM-induced keratinocyte cell death. Furthermore, NM increased transient receptor potential vanilloid 1 (TRPV1) expression, intracellular Ca^2+^ content, and the activities of Ca^2+^/calmodulin-dependent kinase kinase β (CaMKKβ), AMP-activated protein kinase (AMPK), unc-51-like kinase 1 (ULK1), and mammalian target of rapamycin (mTOR). NM-induced autophagy in keratinocytes was abolished by treatment with inhibitors of TRPV1 (capsazepine), CaMKKβ (STO-609), AMPK (compound C), and ULK1 (SBI-0206965) as well as *TRPV1*, *CaMKKβ*, and *AMPK* siRNA transfection. In addition, an mTOR inhibitor (rapamycin) had no significant effect on NM-stimulated autophagy or cell death of keratinocytes. Finally, the results of the in vivo experiment in NM-treated skin tissues were consistent with the findings of the in vitro experiment. In conclusion, NM-caused dermal toxicity by overactivating autophagy partially through the activation of TRPV1-Ca^2+^-CaMKKβ-AMPK-ULK1 signaling pathway. These results suggest that blocking TRPV1-dependent autophagy could be a potential treatment strategy for NM-caused cutaneous injury.

## Introduction

Nitrogen mustard (NM) is a chemical alkylating agent that was first used as a chemical warfare agent in World War I. Based on their alkylating effects, many kinds of NM derivatives, including mechlorethamine, chlorambucil, and melphalan have been widely used in the clinic against various tumors, such as lymphoma, leukemia, and multiple myeloma.^1^ However, the clinical utility of NM is limited by its dose-dependent side effects especially cutaneous toxicity.^1^ NM inflicts incapacitating skin injuries characterized by blistering, inflammation, induration, and edema.^2,3^ There is currently no effective therapy for NM-caused dermal toxicity, due to a lack of understanding of the associated mechanism(s).

Autophagy, a cellular pathway involved in protein and organelle degradation, has been found to be involved in many pathophysiological conditions, including skin disorders.^4^ It has been demonstrated that ultraviolet (UV) irradiation induces autophagy and autophagy- and lysosomal-related gene expression in the epidermis thereby promoting skin tumor growth and progression.^5,6^ Recently, it has been found that mitophagy is induced by trifloxystrobin (a strobilurin class fungicide), which plays an important role in trifloxystrobin-caused cytotoxicity in human skin keratinocytes.^7^ These results indicate a linkage between autophagy and cutaneous diseases associated with physical or chemical exposure. Moreover, BO-1051 (an NM derivative) has been shown to induce autophagy and inhibition of autophagy significantly augmented the cytotoxic effect of BO-1051 in human glioma cells,^8^ suggesting a protective effect of autophagy against NM-induced cytotoxicity. However, limited information is available regarding the potential contribution of autophagy to NM-induced dermal toxicity and the underlying mechanisms.

Transient receptor potential vanilloid 1 (TRPV1), a member of a nonselective cationic channel family, regulates the influx of Ca^2+^. TRPV1 is widely expressed in various tissues, including the brain,^9^ kidney,^10^ bronchial epithelial cells,^11^ and even in keratinocytes in the epidermis.^12^ Accumulating evidence indicates that TRPV1 can be directly activated by vanilloids, UV radiation, heat or protons (reduced pH), and conditions that occur during tissue injury,^13^ thus implicating the channel as a primary cellular sensor of thermal or chemical stimulation in the skin. Previous studies have shown that TRPV1 plays a key role in UV-induced skin damage.^14,15^ However, the role of TRPV1 in NM-induced dermal toxicity is not clear.

AMP-activated protein kinase (AMPK) is a major metabolic energy sensor that regulates energy homeostasis and metabolic stress by controlling several homeostatic mechanisms, including autophagy and protein degradation.^16,17^ AMPK serves as a positive regulator of autophagy mainly by inhibiting the mammalian target of rapamycin (mTOR) complex and phosphorylating unc-51-like kinase 1 (ULK1, the mammalian ortholog of Atg1).^16^ Reportedly, an increase in intracellular Ca^2+^ serves as a potent inducer of autophagy by activating the Ca^2+^/calmodulin-dependent kinase kinase β (CaMKKβ)-AMPK signaling pathway.^17^ Moreover, researchers have demonstrated that activation of TRPV1 causes an influx of Ca^2+^, which activates AMPK-induced autophagy, thereby reducing CD4^+^CD8^+^ thymocyte apoptosis, and impeding foam cell formation in oxLDL-treated vascular smooth muscle cells as well as promoting cancer cell death.^18–20^ These results suggest that AMPK-mediated autophagy might play an important role in the physiological function of TRPV1. Accordingly, in the current study, we investigated the role of autophagy in NM-induced dermal toxicity as well as the potential involvement of the TRPV1-Ca^2+^-CaMKKβ-AMPK signaling pathway in cultured HaCaT cells in vitro and in the skin from SKH-1 hairless mice in vivo.

Our results demonstrated, for the first time, that NM-caused dermal toxicity by overactivating autophagy partially through the activation of the TRPV1-Ca^2+^-CaMKKβ-AMPK-ULK1 signaling pathway. These results provide new insight into the mechanism of NM-induced dermal toxicity and indicate that blocking TRPV1-dependent autophagy could be a potential treatment strategy for NM-caused cutaneous injury.

## Results

### NM-induced keratinocyte cell death

**Fig. 1:**
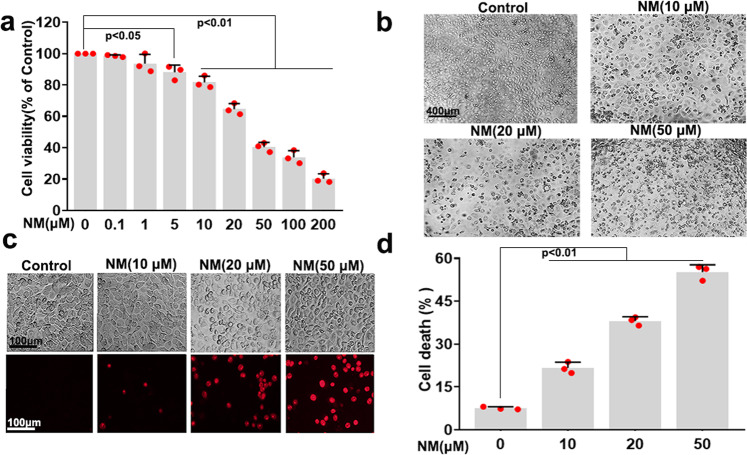
NM-induced keratinocyte cell death. **a** Cells were treated with different concentrations of NM (0.1, 1, 5, 10, 20, 50, 100, and 200 μM) for 24 h. Subsequently, cell viability was measured using a CCK-8 detection kit. **b** Cells were treated with NM (10, 20, and 50 μM) for 24 h, and the morphology of cells was detected by microscopy. Cells were treated with NM (10, 20, and 50 μM) for 24 h, and cell death was determined with PI staining followed by **c** fluorescence microscopy or **d** flow cytometry. Values are presented as means ± SD (*n* = 3)

Histopathology of skin lesions reveals that NM targets basal epidermal keratinocytes in skin tissue and causes cell death.^21^ Therefore, we used human HaCaT keratinocytes as an in vitro NM exposure model. We found that treatment with different concentrations (0.1, 1, 5, 10, 20, 50, 100, and 200 μM) of NM for 24 h significantly inhibited cell viability in a dose-dependent manner, and that the LC50 of NM was 33.94 μM in keratinocytes (Fig. 1a). As shown in Fig. 1b, NM robustly changed the morphology of keratinocytes with irregular cell shape, weakened cell membrane refractive index; cell debris and dead cells were visible. Moreover, with PI staining, we also found that NM markedly increased keratinocyte cell death (Fig. 1c, d and Supplementary Fig. S1). These results suggested that NM dose-dependently caused keratinocyte cell death. Accordingly, 20 μM NM (resulting in ~40% decrease in cell viability) was used in subsequent experiments to clarify the underlying mechanism of NM-induced dermal toxicity.

### NM-induced autophagy in keratinocytes

**Fig. 2:**
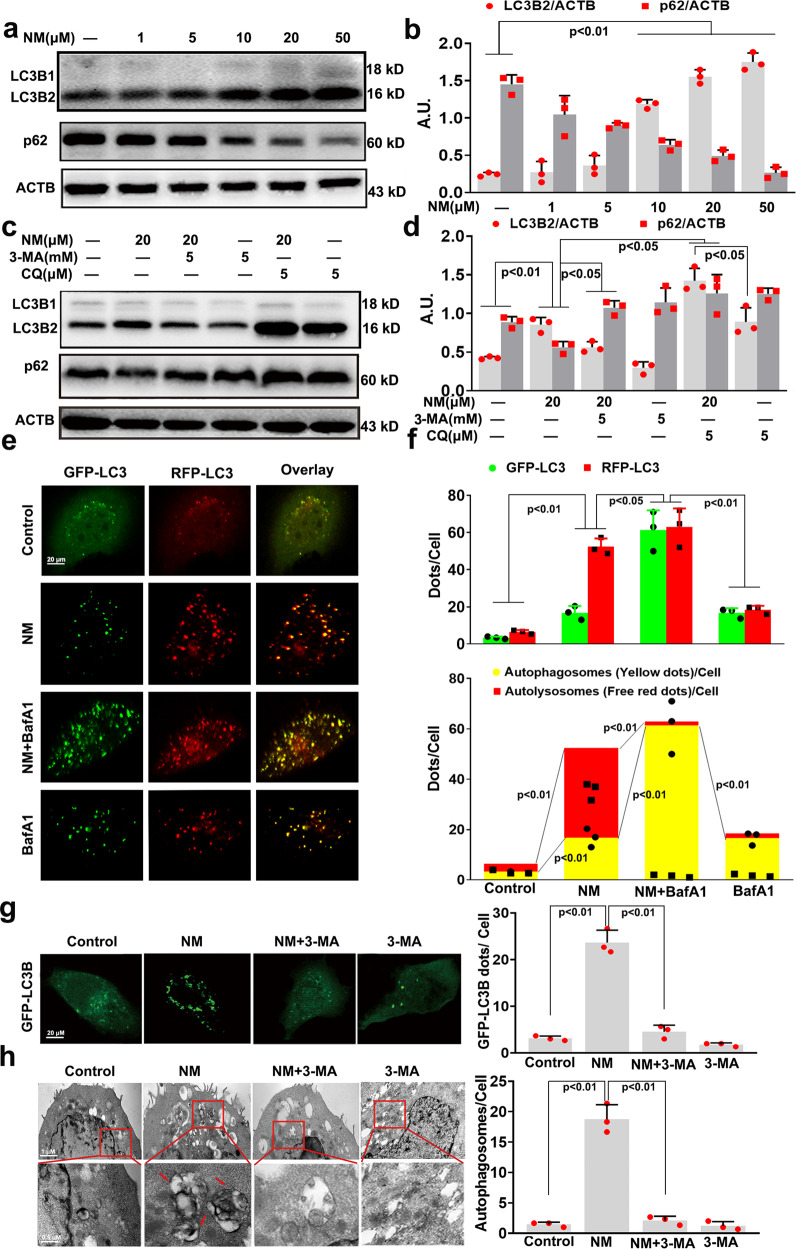
NM-induced autophagy in keratinocytes. **a** Cells were treated with NM at a series of concentrations (1, 5, 10, 20, and 50 µM) for 24 h. The levels of LC3B2 and p62 were detected by western blot analysis. **b** The bar graphs show the quantification of the indicated proteins. **c** Cells were pretreated with 3-MA (5 mM) or CQ (5 µM) for 1 h, then exposed to NM (20 µM) for another 24 h. Total cell lysates were immunoblotted with anti-p62, anti-LC3B, and anti-ACTB antibodies. **d** The bar graphs show the quantification of LC3B2 and p62. **e** Cells were transfected with RFP-GFP-LC3B for 24 h, subjected to BafA1 (10 nM) for 1 h, then exposed to NM (20 µM) for another 24 h. Representative images of fluorescent LC3 puncta are shown. **f** Mean numbers of GFP and RFP dots/cell (top). Mean number of autophagosomes (yellow dots in merged images) and autolysosomes (red dots in merged images) /cell (bottom). **g** Keratinocytes were transfected with a plasmid expressing GFP-LC3B. After 24 h, the cells were preincubated with 3-MA (5 mM) for 1 h. Then, cells were incubated with NM (20 µM) for an additional 24 h. Following fixation, cells were immediately visualized by confocal microscopy and the number of GFP-LC3B dots in each cell was counted. **h** Keratinocytes were pretreated with 3-MA (5 mM) for 1 h, followed by treatment with NM (20 µM) for another 24 h. Then, autophagosomes were detected by transmission electron microscopy as described in the Materials and Methods section. Arrows indicate the autophagosomes. Values are expressed as the mean ± SD (*n* = 3); A.U. arbitrary units

The ratio of light chain 3 beta 2 (LC3B2) to β-actin (ACTB) is an important indicator of autophagy.^22^ NM (≥10 μM) was found to increase the ratio of LC3B2 to ACTB compared to the control and decrease the level of sequestosome 1 (SQSTM1/p62) (Fig. 2a, b). 3-methyladenine (3-MA, an inhibitor of early autophagy stages^22^), markedly inhibited NM-induced LC3 conversion and p62 degradation (Fig. 2c, d). To monitor autophagic flux, LC3B2 levels were measured in the presence of chloroquine (CQ), which inhibits autophagosome-lysosome fusion.^22^ CQ resulted in a greater accumulation of LC3B2 in keratinocytes after incubation with NM (20 µM) for 24 h compared to that in cells treated with CQ alone (Fig. 2c, d). Moreover, tandem fluorescent red fluorescent protein (RFP)- green fluorescent protein (GFP)-LC3 staining is another useful tool for distinguishing autophagy pathway intermediates, which allows one to evaluate the extent of autophagosome and autolysosome formation simultaneously, because LC3 puncta labeled with both GFP and RFP represent autophagosomes, whereas those labeled with RFP alone represent autolysosomes.^23^ As shown in Fig. 2e, f, NM (20 µM) notably increased the numbers of GFP and RFP dots per cell. In the merged images, more free red dots than yellow dots were seen, indicating markedly induced autolysosome formation compared with autophagosomes, and suggesting that NM increased autophagic flux. Meanwhile, bafilomycin A1 (BafA1), which inhibits the acidification of organelles and, subsequently, autophagosome-lysosome fusion,^24,25^ challenge resulted in further accumulation of autophagosomes in NM-treated keratinocytes compared to cells treated with BafA1 alone (Fig. 2e, f). These results suggested that NM promoted cellular autophagic flux in keratinocytes.

To further characterize NM-induced autophagy in keratinocytes, cells were transfected with the GFP-LC3B plasmid, a specific marker of phagophores and autophagosomes, and visualized by fluorescence microscopy.^22^ NM (20 µM) notably increased the number of GFP-LC3B dots compared with the control group, which then decreased in the presence of 3-MA (Fig. 2g). Moreover, transmission electron microscopy, the most valid method for both qualitative and quantitative analysis of autophagy,^22^ showed that more vacuoles were present in NM-treated keratinocytes, but the number decreased when the cells were pretreated with 3-MA (Fig. 2h). These results suggested that NM treatment upregulated the entire autophagy process in keratinocytes.

### The overactivation of autophagy contributed to NM-induced keratinocyte cell death

**Fig. 3:**
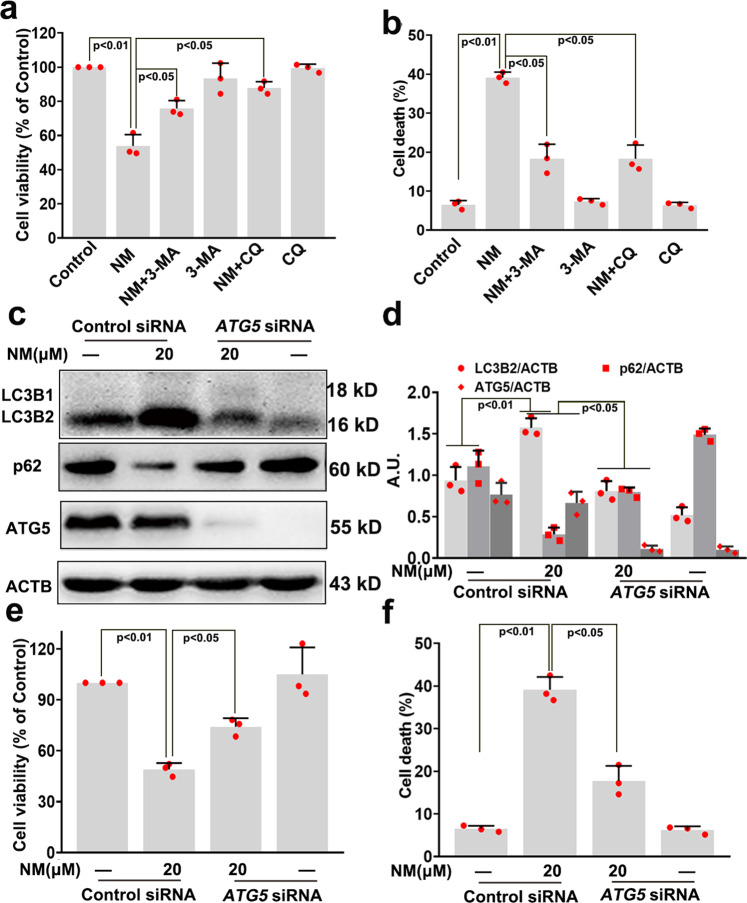
The overactivation of autophagy contributed to NM-induced keratinocyte cell death. Cells were pretreated with 3-MA (5 mM) and CQ (5 µM) for 1 h, followed by treatment with NM (20 µM) for another 24 h. **a** Cell viability was measured by a CCK-8 detection kit and **b** cell death was detected with PI staining by flow cytometry. **c** ATG5 was knocked down by *ATG5* siRNA as described in “Materials and methods”. At 24-h post-transfection, cells were treated with NM (20 µM) for 24 h. Cells were collected and lysed, and then western blot analysis was performed. **d** Bar charts show the quantification of the indicated proteins. Cells were treated as described in (**c**). Then (**e**) cell viability was measured by a CCK-8 kit and (**f**) Cell death was detected by PI staining followed by flow cytometry. Values are expressed as the mean ± SD (*n* = 3); A.U. arbitrary units

3-MA, CQ, and small interfering RNA (siRNA) targeting *ATG5* were used to investigate the role of autophagy in NM-caused keratinocyte cell death. As shown in Fig. 3a–f and Supplementary Fig. S1, 3-MA and CQ as well as *ATG5* siRNA transfection ameliorated NM-induced keratinocyte cell death. In addition, 3-MA, CQ and *ATG5* siRNA had no obvious effects on keratinocyte cell viability (Fig. 3a–f and Supplementary Fig. S1). These results indicated that autophagy contributed to the cytotoxicity of NM in keratinocytes. In 2013, Levine et al.^26^ described “autosis” as a subtype of autophagy-dependent cell death, which is a Na^+^, K^+^-ATPase–regulated form of cell death. It has been demonstrated that autosis can be inhibited by digoxin (a Food and Drug Administration [FDA]-approved antagonist of Na^+^, K^+^-ATPase); meanwhile, BafA1 does not reduce autosis. Therefore, BafA1 and digoxin were further used to investigate the role of autosis in NM-induced keratinocyte cell death. As shown in Supplementary Fig. S2, BafA1 pretreatment significantly attenuated NM-induced cell death of keratinocytes; in contrast, digoxin had no effect on NM-caused keratinocyte cell death. These results indicated that NM-induced autophagic cell death of keratinocytes, which was not autosis.

### NM-induced autophagy via the AMPK-ULK1 pathway in keratinocytes

**Fig. 4:**
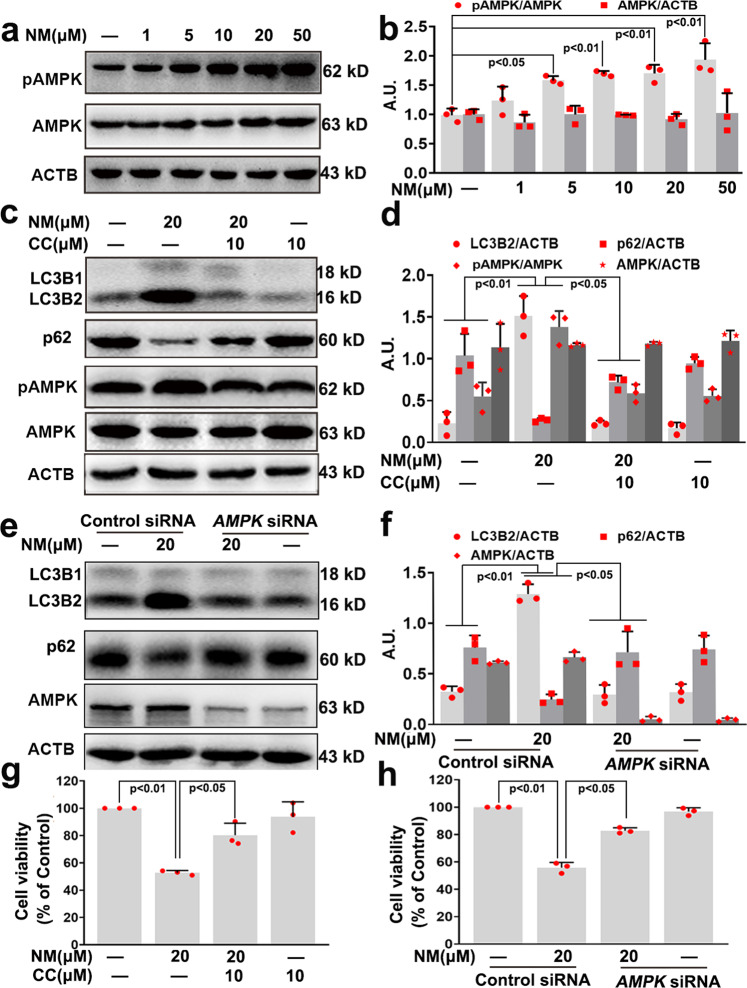
NM-induced autophagy through AMPK activation in keratinocytes. **a** Cells were treated with NM at a series of concentrations (1, 5, 10, 20, and 50 µM) for 24 h. AMPK and pAMPK expression were measured by western blotting. **b** The bar graphs show the quantification of endogenous pAMPK. **c** Cells were pretreated with CC (10 µM) for 1 h followed by exposure to NM (20 µM) for an additional 24 h. Cells were collected and lysed, and then western blot analysis was performed. **d** The bar graph shows the quantification of the indicated proteins. **e** AMPK was knocked down by *AMPK* siRNA as described in “Materials and methods”. At 24-h post-transfection, the cells were treated with NM (20 µM) for 24 h. Cells were collected and lysed, then western blot analysis was performed. **f** The bar graphs show the quantification of the indicated proteins. **g** Cells were treated as described in (**c**), and cell viability was detected by a CCK-8 detection kit. **h** Cells were treated as described in (**e**), and cell viability was detected by a CCK-8 detection kit. Values are expressed as the means ± SD (*n* = 3); A.U. arbitrary units

Previous studies have demonstrated that AMPK is a key regulator of autophagy.^16^ Our results showed that NM resulted in a dose-dependent AMPK activation, as monitored by AMPK phosphorylation (Fig. 4a, b). Compound C (CC, a potent AMPK inhibitor) abolished NM-induced AMPK activation and caused a decrease in autophagy (Fig. 4c, d). Meanwhile, NM-stimulated autophagy was also diminished by *AMPK* siRNA in keratinocytes (Fig. 4e, f). In addition, CC and *AMPK* siRNA treatment notably inhibited NM-caused cell death of keratinocytes (Fig. 4g, h). Together, these results indicated that AMPK inhibition suppressed autophagy thereby attenuating NM-caused cell death of keratinocytes.

**Fig. 5:**
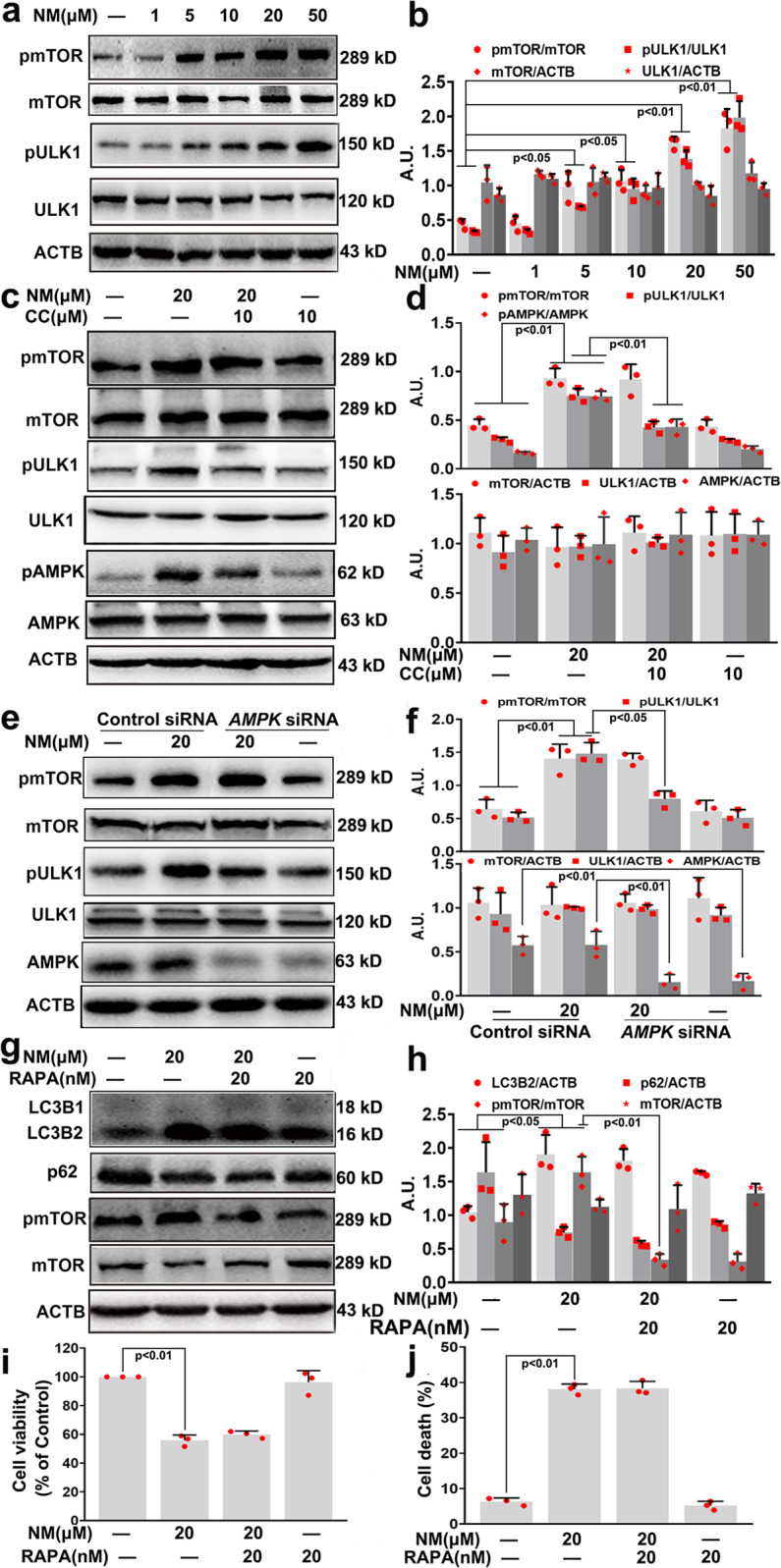
NM-activated ULK1 and mTOR in keratinocytes. **a** Cells were treated with NM at a series of concentrations (1, 5, 10, 20, and 50 µM) for 24 h. The expression of mTOR, pmTOR, ULK1, and pULK1 was measured by western blotting. **b** The bar graphs show the quantification of endogenous pmTOR and pULK1. **c** Cells were pretreated with CC (10 µM) for 1 h followed by exposure to NM (20 µM) for an additional 24 h. Total cell lysates were immunoblotted with anti-mTOR, anti-pmTOR, anti-ULK1, anti-pULK1, anti-pAMPK, anti-AMPK, and anti-ACTB antibodies. **d** The bar graph shows the quantification of the indicated proteins. **e** AMPK was knocked down by *AMPK* siRNA as described in “Materials and methods”. At 24-h post-transfection, the cells were treated with NM (20 µM) for 24 h. Cells were collected and lysed, then western blot analysis was performed. **f** The bar graphs show the quantification of the indicated proteins. **g** Cells were pretreated with RAPA (20 nM) for 1 h followed by exposure to NM (20 µM) for an additional 24 h. Total cell lysates were immunoblotted with anti-mTOR, anti-pmTOR, anti-LC3, anti-p62, and anti-ACTB antibodies. **h** The bar graph shows the quantification of the indicated proteins. **i** Cell viability was detected by a CCK-8 detection kit. **j** Cell death was measured with PI staining by flow cytometry. Values are expressed as the means ± SD (*n* = 3); A.U. arbitrary units

Since AMPK can activate autophagy through the activation of ULK1 or by inhibiting mTOR signaling,^16^ we next examined alterations in the AMPK/ULK1 and mTOR pathways induced by NM. As shown in Fig. 5a, b, the phosphorylation of ULK1 and mTOR was increased by NM in a dose-dependent manner. AMPK inhibition or knockdown significantly abolished NM-induced increase of pULK1 level, however, had no obvious effect on NM-induced pmTOR expression in keratinocytes (Fig. 5c–f). Moreover, inhibition of ULK1 by SBI-0206965 (SBI) significantly prevented NM-induced autophagy and restored cell viability following NM treatment (Supplementary Fig. S3a, d). Although the level of pmTOR was increased by NM, the mTOR inhibitor rapamycin (RAPA) had no obvious effect on NM-induced LC3 conversion or p62 degradation, indicating that NM-mediated autophagy activation occurred independently of mTOR regulation in keratinocytes (Fig. 5g, h). In addition, RAPA treatment had no effect on NM-caused cell death or ULK1 activation in keratinocytes (Fig. 5i, j and Supplementary Fig. S4a, b). Thus, we concluded that the AMPK-ULK1 pathway was required for NM-induced autophagy and keratinocyte cell death.

### NM-activated AMPK via the Ca^2+^-CaMKKβ pathway in keratinocytes

**Fig. 6:**
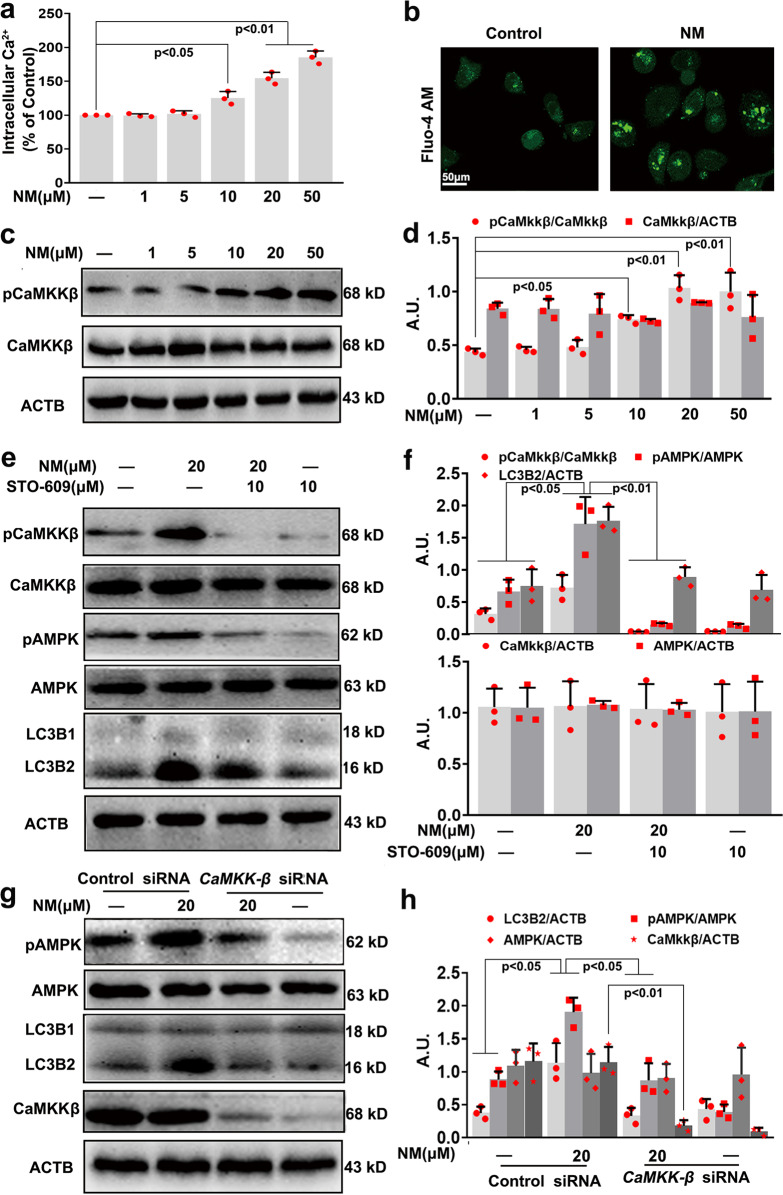
NM-activated AMPK via the Ca^2+^-CaMKKβ pathway in keratinocytes. **a** Cells were treated with NM at a series of concentrations (1, 5, 10, 20, and 50 µM) for 24 h. The intracellular Ca^2+^ content was measured with Fluo-4 AM by an Infinite^TM^ M200 Microplate Reader, according to the manufacturer’s instructions. **b** Representative images of intracellular Ca^2+^ detected with a Radiance 2000 laser scanning confocal microscope. **c** Cells were treated as described in (**a**) and the expression of pCaMKKβ and CaMKKβ was measured by western blotting. **d** The bar graphs show the quantification of endogenous pCaMKKβ. **e** Cells were pretreated with STO-609 (10 µM) for 1 h followed by exposure to NM (20 µM) for an additional 24 h. Cells were collected and lysed, then western blot analysis was performed. **f** The bar graphs show the quantification of the indicated proteins. **g** CaMKKβ was knocked down by *CaMKKβ* siRNA as described in “Materials and methods”. At 24-h post-transfection, the cells were treated with NM (20 µM) for 24 h. Cells were collected and lysed, then western blot analysis was performed. **h** The bar graphs show the quantification of the indicated proteins. Values are expressed as the means ± SD (*n* = 3); A.U. arbitrary units

In view of the finding that the Ca^2+^-CaMKKβ pathway plays an important role in the regulation of AMPK-mediated autophagy.^27,28^ Therefore, the effects of NM on Ca^2+^ influx and CaMKKβ activity were investigated. As shown in Fig. 6a, b, NM significantly increased the intracellular Ca^2+^ content. NM-induced a significant increase of pCaMKKβ expression in a dose-dependent manner (Fig. 6c, d), indicating the activation of CaMKKβ by NM. Moreover, STO-609 (a CaMKK-α/β inhibitor) treatment markedly inhibited NM-induced AMPK and ULK1 activation and autophagy induction, ultimately attenuating NM-induced cell death (Fig. 6e, f and Supplementary Fig. S3b, d). In addition, STO-609 had no obvious effect on NM-induced pmTOR expression in keratinocytes (Supplementary Fig. S3b). Meanwhile, *CaMKK-*β siRNA also abolished the activation of AMPK and the induction of autophagy in NM-stimulated keratinocytes (Fig. 6g, h). Additionally, it has been demonstrated that AMPK monitors the cellular energy status which is usually stimulated by inhibition of mitochondrial ATP synthesis.^29^ Accordingly, ATP levels were detected in NM-treated keratinocytes, we found that NM slightly but not significantly increased ATP levels (Supplementary Fig. S5a), indicating that NM-activated AMPK in an ATP independent manner in keratinocytes. These results suggested that the Ca^2+^-CaMKKβ pathway was required for AMPK activation and subsequent NM-induced autophagy in keratinocytes.

### NM-induced Ca^2+^ influx in a TRPV1-dependent manner in keratinocytes

**Fig. 7:**
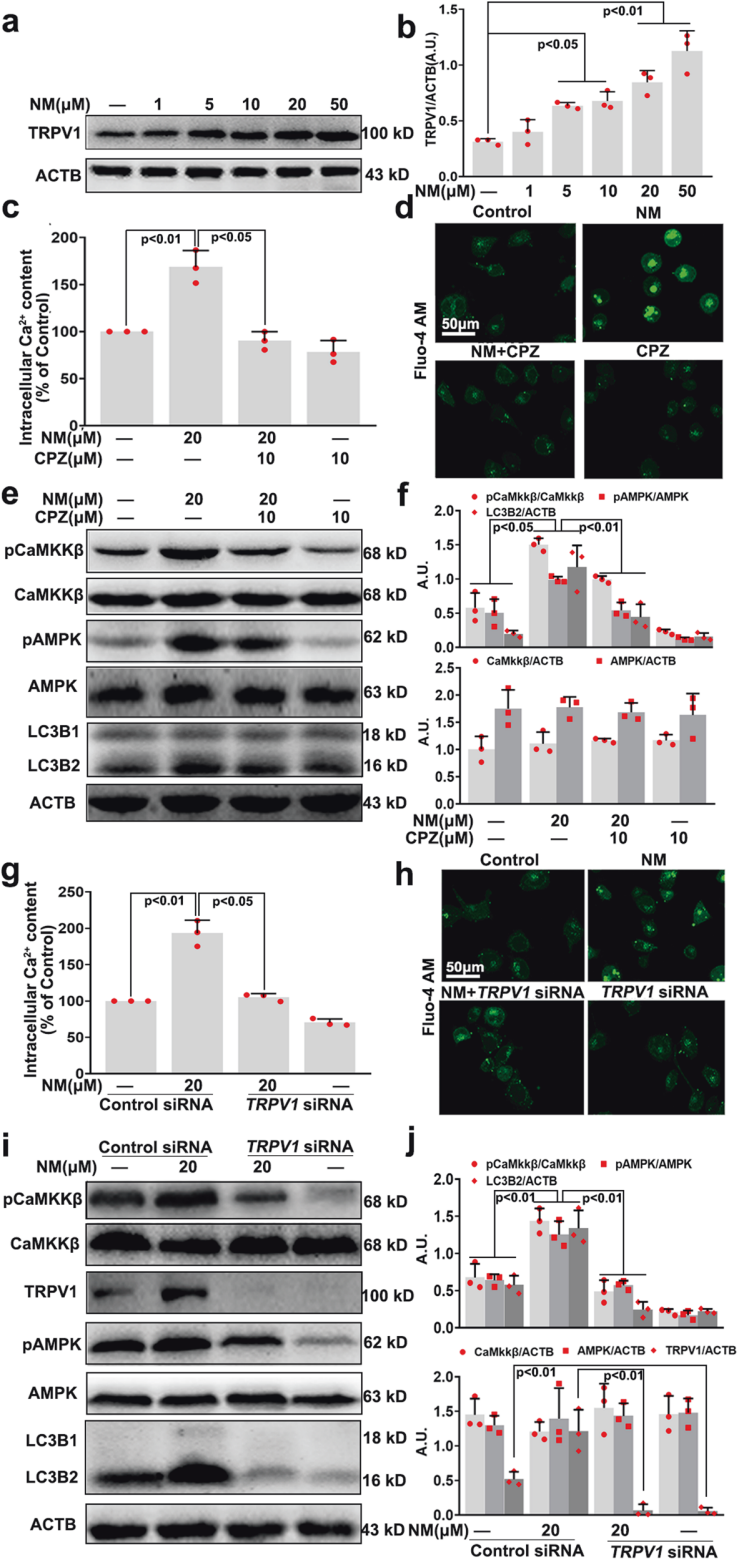
NM-induced Ca^2+^ influx in a TRPV1-dependent manner in keratinocytes. **a** Cells were treated with NM at a series of concentrations (1, 5, 10, 20, and 50 µM) for 24 h. The expression of TRPV1 was detected by western blotting. **b** The bar graphs show the quantification of TRPV1. Cells were pretreated with CPZ (10 µM) for 1 h followed by exposure to NM (20 µM) for an additional 24 h. **c** The intracellular Ca^2+^ content was measured with Fluo-4 AM using an Infinite^TM^ M200 Microplate Reader, according to the manufacturer’s instructions. **d** Representative images of intracellular Ca^2+^ detected with a Radiance 2000 laser scanning confocal microscope. **e** Cells were collected and lysed for western blot analysis. **f** The bar graphs show the quantities of the indicated proteins. TRPV1 was knocked down by *TRPV1* siRNA as described in the “Materials and methods” section. At 24-h post-transfection, the cells were treated with NM (20 µM) for 24 h. **g** The intracellular Ca^2+^ content was measured with Fluo-4 AM using an Infinite^TM^ M200 Microplate Reader, according to the manufacturer’s instructions. **h** Representative images of intracellular Ca^2+^ detected with a Radiance 2000 laser scanning confocal microscope. **i** Cells were collected and lysed for western blot analysis. **j** The bar graphs show the quantities of the indicated proteins. Values are expressed as the means ± SD (*n* = 3); A.U. arbitrary units

TRPV1 is a non-selective ion channel that regulates the influx of Ca^2+^ and is considered as a primary cellular sensor of thermal and chemical stimulation in the skin.^13^ Thus, the involvement of TRPV1 in the NM-induced increase of intracellular Ca^2+^ content was also explored. As shown in Fig. 7a, b, NM significantly induced TRPV1 expression. The TRPV1 antagonist capsazepine (CPZ) notably inhibited NM-stimulated Ca^2+^ influx and CaMKKβ activation, thereby, inhibiting AMPK and ULK1 activation; meanwhile, CPZ also inhibited NM-induced autophagy and cell death of keratinocytes (Fig. 7c–f and Supplementary Fig. S3c, d). When TRPV1 was knocked down by *TRPV1* siRNA transfection, similar results were found (Fig. 7g–j). These data clearly indicated that TRPV1 was required for Ca^2+^ accumulation and the subsequent activation of the CaMKKβ-AMPK-autophagy signaling pathway induced by NM in keratinocytes.

As NM-stimulated Ca^2+^ influx, the effect of NM on lysosomal function and reactive oxygen species (ROS) generation was investigated. LysoSensor Green DND-189 dye is an acidotropic probe that accumulates in acidic organelles, such as lysosomes, and exhibits a pH-dependent increase in fluorescence intensity upon acidification.^30^ NM increased the LysoSensor Green DND-189 fluorescence intensity (Supplementary Fig. S5b), suggesting lysosomal pH alteration in keratinocytes. As shown in Supplementary Fig. S5c–e, NM induced the expression of the lysosomal-associated membrane protein 1 (LAMP1) and cathepsin D (CTSD) and increased the fluorescence intensity of cells stained by LysoTracker Green (LTG) fluorescent dye, which stains acidic compartments, particularly lysosomes.^31^ In addition, ROS levels were detected by dichloro-dihydro-fluorescein diacetate (DCFH-DA) staining, and we found that NM markedly increased ROS levels in keratinocytes (Supplementary Fig. S5f). These results suggested that NM both induced lysosomal activity and increased ROS levels in keratinocytes. However, CPZ pretreatment abolished NM-induced lysosomal activity and increase of ROS levels (Supplementary Fig. S5b–f). Together, these results, combined with the above data, collaboratively demonstrate that TRPV1 is a potential target for the treatment of NM-caused dermal toxicity.

### NM-induced autophagy via activating the TRPV1 signaling pathway in the skin of SKH-1 hairless mice

**Fig. 8:**
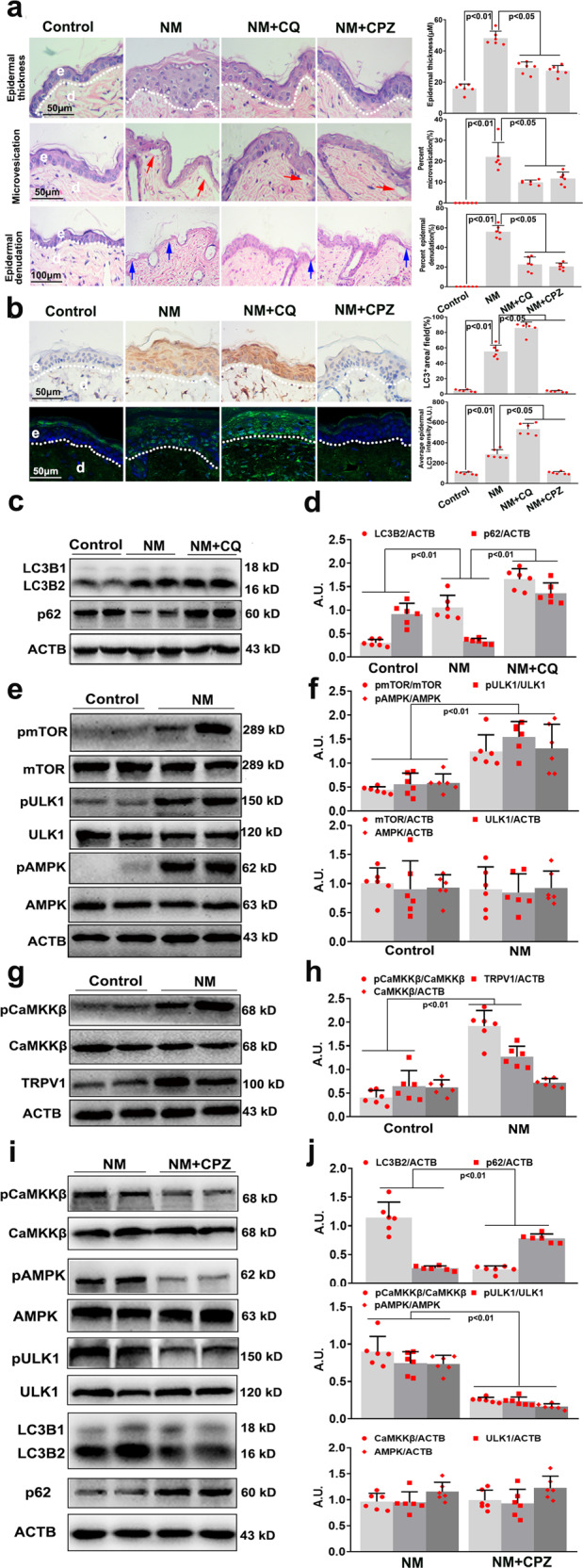
NM-induced autophagy via activating the TRPV1 signaling pathway in the skin of SKH-1 hairless mice. The dorsal skins of 6-week-old male SKH-1 hairless mice with 24 g~26 g weighing (*n* = 6 per group) were treated with CQ (60 mg/kg, i.p.) or CPZ (5 mg/kg, i.p.), they were exposed to 3.2 mg NM in 200 μL acetone as described in the Materials and Methods. Mice were sacrificed at 24 and 72 h following NM exposure, and dorsal skin tissue was collected and fixed in 10% formalin for histopathological analysis or snap frozen in liquid nitrogen for western blot analysis. **a** At 24 h or 72 h after NM exposure, skin wounds and nearby tissues were collected and H&E staining was performed for analysis of epidermal thickness (top), microvesication (middle), and epidermal denudation (bottom). e, epidermis; d, dermis; red arrows, microvesication; blue arrows, epidermal denudation. **b** At 24 h after NM exposure, the skin wounds and nearby tissues were collected and LC3B expression was detected by immunohistological analysis (top) and immunofluorescence analysis (bottom). e, epidermis; d, dermis. The skin tissues were harvested at 72-h post NM exposure and lysed, and western blot analysis was performed. **c** The formation of LC3B2 and the degradation of p62 were measured. **d** The bar graphs show the quantification of LC3B2 and p62. **e** The levels of mTOR, pmTOR, ULK1, pULK1, pAMPK, and AMPK were measured. **f** The bar graphs show the quantification of the indicated proteins. **g** The expression of TRPV1, pCaMKKβ, and CaMKKβ was measured. **h** The bar graphs show the quantification of the indicated proteins. **i** The expression of the indicated protein was detected by western blotting. **j** The bar graphs show the quantification of indicated proteins. Values are expressed as the means ± SD (*n* = 6); A.U. arbitrary units

We additionally investigated NM-induced dermal toxicity in skin in vivo. Epidermal thickness, microvesication (epidermal-dermal separation), and epidermal denudation have been considered as the primary injury end points in NM-exposed dorsal mouse skin.^3^ In accordance with previous observations, NM significantly increased skin epidermal thickness in post 24-h exposure and microvesication and epidermal denudation in skin post 72-h exposure, which were significantly attenuated by CQ treatment (Fig. 8a). Accumulating evidence has revealed that NM causes a strong inflammatory response in skin;^32^ therefore, the effect of CQ on NM-induced inflammation was determined in vivo. As expected, CQ significantly decreased NM-caused cyclooxgenase 2 (COX2) and matrix metalloproteinase 9 (MMP9) expression in skin post 72-h exposure (Supplementary Fig. S6a, b). CQ also inhibited NM-induced autophagy in skin (Fig. 8b–d). As shown in Fig. 8e–h, NM increased the levels of TRPV1, pCaMKKβ, pAMPK, pULK1, and pmTOR, indicating that the TRPV1 signaling pathway was activated in NM-exposed skin. Moreover, CPZ treatment abolished the effect of NM on the TRPV1 signaling pathway thereby attenuating NM-induced autophagy, which, subsequently, ameliorated NM-caused skin injury (Fig. 8a–j and Supplementary Fig. S6a, b). The results suggested that NM-induced autophagy via activating the TRPV1 signaling pathway, ultimately, causing cutaneous injury in vivo.

## Discussion

NM causes severe skin injury, which lacks effective targeted therapies. The major limitation is that the specific mechanism of NM-induced injury is not thoroughly understood. Here, for the first time, we found that NM-caused dermal toxicity via overactivating autophagy.

Autophagy is a cellular housekeeping process that helps to maintain cellular homeostasis by removing dysfunctional or damaged intracellular organelles.^33^ When it functions properly, autophagy may aid in cell survival when cells undergo stimulation and stress.^34^ However, excessive autophagic activation evokes autophagic programmed cell death.^35^ Recently, autosis has been identified as a subtype of autophagy-dependent cell death, that occurs with high autophagic activity, in the absence of apoptotic and necrotic markers and yet is not fully regulated by typical autophagy markers.^36^ Autosis is regulated by Na^+^, K^+^-ATPase, which can be inhibited by digoxin; however, BafA1 has no effect on autosis, suggesting that this form of cell death does not require late stages of autophagy.^26^ Thus, we investigated whether NM-induced autosis in keratinocytes. We found that BafA1 significantly attenuated NM-induced keratinocyte cell death; however, digoxin had no obvious effect on this process (Supplementary Fig. S2). Moreover, autosis is morphologically characterized by enhanced cell-substrate adherence, fragmented or vanished ER structure, focal swelling of the perinuclear space, and mild chromatin condensation,^26^ which were not found in NM-treated keratinocytes (Fig. 2H). These data indicated that NM-induced autophagic cell death of keratinocytes, which was not autosis. It has been demonstrated that downregulation of autophagy leads to an accumulation of misfolded proteins in keratinocytes, which has been associated with the development of skin disorders.^37–39^ Several studies have implicated autophagy as a protective mechanism during the development and progression of skin diseases, and pharmacological approaches have recently been developed to prevent the pathogenesis of skin diseases through autophagy induction.^40^ In contrast, another study found that UV radiation induces autophagy in the skin, and that epidermis-specific inhibition of ATG7 and p62 protects against UV-induced inflammation and skin tumorigenesis.^5,6^ Recently, trifloxystrobin, a strobilurin class fungicide, was also found to induce mitophagy via mitochondrial damage in keratinocytes.^7^ These findings indicate a complex role of autophagy in maintaining homeostasis in the epidermis under physical or chemical exposure. Our data showed that NM induced an excessive autophagic process that contributed to NM-induced cell death of keratinocytes (Figs. 2a–h, 3a–f, 8a–d, and Supplementary Fig. S1). This work provides new evidence that autophagy contributes substantially to NM-mediated dermal toxicity, suggesting that targeting autophagy may have therapeutic potential for NM-induced cutaneous injury.

Meanwhile, NM has also been widely used as a chemotherapeutic agent. Previous studies have reported that chemotherapy can induce cytoprotective autophagy, which promotes therapeutic resistance. Pharmacological inhibitors or siRNAs that inhibit autophagy can sensitize resistant cancer cells to conventional chemotherapies and specifically target autophagy-addicted tumors.^41^ Therefore, CQ (an FDA-approved drug that interferes with autophagy) has been widely used in phase I and II clinical trials in combination with several standard chemotherapies. However, overall the outcomes of these trials have not been encouraging,^42^ suggesting that the picture remains quite indistinct when we consider the potential for manipulation of chemotherapy-induced autophagy for therapeutic benefit. It has been demonstrated that inhibition of autophagy enhances BO-1051 (an NM derivative)-induced cell death in cancer cells,^8,43,44^ suggesting the cytoprotective role of autophagy in NM’s anti-tumor effect. However, the exact role of autophagy in the anti-tumor effect of NM needs further elucidation, which might be dependent on biological factors and technical aspects.

Furthermore, the potential mechanisms of NM-induced autophagy were also investigated. Autophagy is critically coordinated by the action of a set of several key components, including ATG, and is regulated by intricate networks, such as mTOR.^45^ NM derivatives have been found to stimulate autophagy via inhibition of the mTOR signaling pathway in cancer cells.^8,46^ In our study, we found that NM-mediated autophagy activation occurred independently of mTOR regulation in keratinocytes. Apart from the classical mTOR-dependent pathway, several mTOR-independent autophagy pathways, such as Ca^2+^, AMPK, miRNA,^45^, and spermidine,^47^ have also been implicated in the regulation of autophagy. Previous studies have indicated that AMPK is a key regulator of autophagy mainly via the inhibition of mTOR and phosphorylation of ULK.^16^ However, researchers have recently reported that inactivation of mTOR is not always necessary for AMPK-mediated autophagy.^48,49^ Consistent with these findings, our results demonstrated that NM-induced autophagy by activating AMPK through ULK1 phosphorylation, independent of mTOR inhibition in keratinocytes (Figs. 4a–h, 5a–j and Supplementary Figs. S3a, d and S4a, b). Reportedly, AMPK, a sensor of cellular energy status, is activated by upstream kinases, including liver kinase B1 (LKB1), and CaMKKβ.^50^ AMPK can be phosphorylated and activated by tumor suppressor kinase LKB1 at low energy levels and by CaMKK-β in response to an increase in intracellular Ca^2+^ content.^51,52^ It has been found that cadmium-mediated ROS generation causes poly (ADP)-ribose polymerase activation and energy depletion, and eventually induces autophagy through the activation of LKB1-AMPK signaling and the downregulation of mTOR in skin epidermal cells.^53^ Furthermore, UVB can stimulate autophagy through AMPK activation in epidermal cells, which provides a new target and strategy for better prevention and treatment of UV-induced skin injury.^54,55^ In the present study, we identified that NM increased intracellular Ca^2+^ levels, but had no significant effect on ATP levels, indicating that NM-activated AMPK in an ATP independent manner in keratinocytes (Fig. 6a–h and Supplementary Figs. S3b and d and S5a). Accordingly, we demonstrated that NM increased the intracellular Ca^2+^ and induced autophagy through activating the CaMKKβ-AMPK signaling pathway in keratinocytes. Although the Ca^2+^-CaMKKβ-AMPK pathway has been found to be involved in autophagy regulation in other cell types, our results, for the first time, demonstrated that the Ca^2+^-CaMKKβ-AMPK-ULK1 signaling pathway is vital for NM-mediated autophagy in keratinocytes. This work provides new insight into the mechanism of autophagy induction by NM.

In addition to AMPK, our results support an important role for TRPV1 in NM-mediated autophagy in keratinocytes. TRPV1 is a non-selective, thermo-sensitive ion channel that belongs to the TRP channel family functioning as detectors of noxious stimuli.^56,57^ It has been demonstrated that TRPV1 is widely expressed in skin tissues especially in keratinocytes,^58^ and plays important role in skin physiology. Kuo-Feng Huang et al.^15^ indicated that UVB irradiation-induced keratinocyte differentiation required Ca^2+^ influx through TRPV1 activation. Yong-mee Lee et al.^59,60^ found that UV-induced MMP1 expression is mediated in part by the activation of TRPV1 and the subsequent Ca^2+^ influx in human keratinocytes, and a TRPV1-specific blocker prevented UV-induced skin responses, which provides new insight into the development of effective therapeutic methods for photoaging. Herein, we found that NM increased TRPV1 expression, which was followed by an increase in intracellular Ca^2+^, as well as activation of CaMKKβ and AMPK (Figs. 7a, b and 8e–h). CPZ pretreatment or *TRPV1* siRNA transfection notably inhibited NM-induced activation of the Ca^2+^-CaMKKβ-AMPK signaling pathway, thereby attenuating autophagy, which ultimately ameliorated NM-induced dermal toxicity both in vitro and in vivo (Figs. 7c–j, 8i, j and supplementary Fig. S3c, d). These results indicated that NM-triggered autophagy was mainly proceeded by the activation of TRPV1 signaling in skin tissues. Given the close association between Ca^2+^, lysosomal function and ROS generation,^61^ the effect of NM on lysosomal function and ROS generation was additionally investigated. We showed that NM-induced lysosomal activity, which might contribute to its effect on autophagic flux (Supplementary Fig. S5b–e). However, the exact mechanisms underlying NM-induced autophagic flux need further elucidation. Meanwhile, NM increased ROS levels in keratinocytes, and CPZ pretreatment notably abolished NM-induced increase of ROS levels and lysosomal activity (Supplementary Fig. S5b–f). Although the exact mechanisms underlying NM-induced ROS generation and lysosomal function remains to be explored, our current results provide substantial evidence that TRPV1 could be a good candidate for the treatment of NM-caused dermal toxicity.

**Fig. 9:**
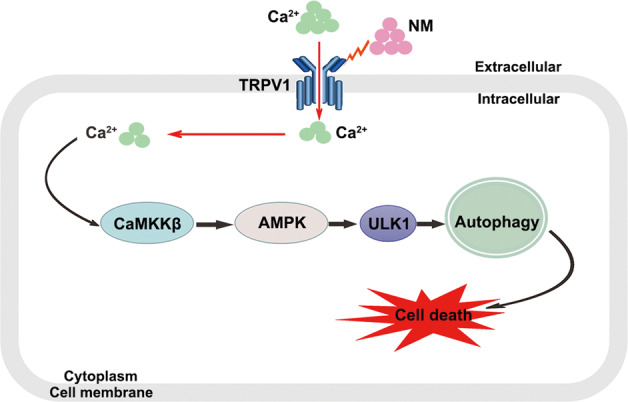
Regulation of NM-induced autophagy in keratinocytes. NM-induced keratinocyte cell death by overactivating autophagy via the proposed signaling pathways: NM induces TRPV1 expression, which is followed by an increase of intracellular Ca^2+^ content, thereby activating CaMKKβ, subsequently increasing AMPK and ULK1 activity and ultimately inducing autophagy

In general, our findings provide a novel mechanism in which NM-induced autophagy overactivation contributes to NM-caused dermal toxicity. Moreover, the TRPV1-Ca^2+^-CaMKKβ-AMPK-ULK1 signaling pathway was found to be important in moderating NM-induced autophagy (Fig. 9). Although it is challenging to discern the extent to which NM causes a robust increase in autophagy flux above the basal level versus an overactivation of autophagy, our current findings still open a new avenue of research regarding the mechanism of NM-induced dermal toxicity, which is an important complement to previous studies. These indicate that targeting TRPV1 or inhibiting autophagy could be effective therapeutic strategies to combat NM-induced skin injury.

## Materials and methods

### Cell treatments

During the logarithmic growth phase, the HaCaT cells were treated with NM (20 μM) for 24 h. All treatments were carried out in a complete culture medium to avoid the induction of autophagy through the serum starvation pathway. Where indicated, cells were also treated with 3-MA (5 mM), CQ (5 µM), BafA1(10 nM), digoxin (0.1 µM), CC (10 µM), STO-609 (10 µM), CPZ (10 µM), SBI (10 µM) or RAPA (20 nM) for 1 h following the addition of NM for another 24 h.

### Animal treatments

About 6-week-old male SKH-1 hairless mice were acclimatized for 1 week prior to NM exposure. The dorsal skin of male SKH-1 hairless mice (n = 6 per group) was exposed to 3.2 mg NM in 200 μL acetone as described before and the control mice received only 200 μL acetone.^62^ CQ (60 mg/kg) or CPZ (5 mg/kg) was intraperitoneally injected into mice at 1 h before NM treatment. The control mice received an equal volume of normal saline. At 4 h after NM exposure, the exposed skin regions were gently wiped with sodium hypochlorite (0.8%) and saline for decontamination. Mice were sacrificed at 24 and 72 h following NM exposure, and the dorsal skin tissue was collected and fixed in 10% formalin for histopathological analysis or snap frozen in liquid nitrogen for western blot analysis. All animal experiments were carried out in strict accordance with the recommendations in the Guide for the Care and Use of Laboratory Animals by the National Institutes of Health and were approved by the Animal Care and Use Committee of the Army Medical University (Chongqing, China; approval no. SYXC-2016-00115).

### Statistical analyses

Quantitative data are presented as the means ± standard deviation (SD) of three experiments. The statistical analysis was conducted with the *t*-test and one-way analysis of variance using SPSS 18.0 statistical software (SPSS Inc., Chicago, IL, USA). A *p*-value < 0.05 was considered statistically significant and the Tukey-Kramer post hoc test was applied if *p* < 0.05.

## Supplementary information

SUPPLEMENTAL MATERIAL

## Data Availability

All data and materials are available to the researchers once published.
