# Supplementary material for: Targeting TRPV1-mediated autophagy attenuates nitrogen mustard-induced dermal toxicity
Source: Signal Transduct Target Ther. 2021 Jan 25;6:29. doi: 10.1038/s41392-020-00389-z (PMC7829253; doi:10.1038/s41392-020-00389-z)
Supplement: Supplementary file 1 — SUPPLEMENTAL MATERIAL [file 41392_2020_389_MOESM1_ESM.doc]

**Supplementary Materials for:**

**Targeting TRPV1-mediated autophagy attenuates nitrogen mustard-induced dermal toxicity**

Mingliang Chen^1,2,3^*, Xunhu Dong^1,3^*, Haoyue Deng^4^*, Feng Ye^1,3^, Yuanpeng Zhao^1,3^, Jin Cheng^1,3^, Guorong Dan^1,3^, Jiqing Zhao^1,3^, Yan Sai^1,3^, Xiuwu Bian^2#^, Zhongmin Zou^1,3#^

*These authors contribute equally to this work

^1^Department of Chemical Defense Medicine, School of Military Preventive Medicine, Third Military Medical University (Army Medical University), 30 Gaotanyan Street, Shapingba District, Chongqing 400038, China.

^2^Institute of Pathology and Southwest Cancer Centre, Southwest Hospital, Third Military Medical University (Army Medical University), Chongqing 400038, China.

^3^Institute of Toxicology, School of Military Preventive Medicine, Third Military Medical University (Army Medical University), 30 Gaotanyan Street, Shapingba District, Chongqing 400038, China.

^4^ State Key Laboratory of Trauma, Burns and Combined Injury, Second Department of Research Institute of Surgery, Daping Hospital, Third Military Medical University (Army Medical University), Chongqing 400042, China.

**#Correspondence**

Xiuwu Bian, M.D., Ph.D., Professor, Director, Institute of Pathology and Southwest Cancer Centre, Southwest Hospital, Third Military Medical University (Army Medical University), Chongqing 400038, China.

TEL: 86-23-68752332; FAX: 86-23-68752332; E-mail: bianxiuwu@263.net

Zhongmin Zou, M.D., Ph.D., Professor, Director, Department of Chemical Defense Medicine, School of Military Preventive Medicine, Third Military Medical University (Army Medical University), 30 Gaotanyan Street, Shapingba District, Chongqing 400038, China.

TEL: 86-23-68771526; FAX: 86-23-68771526; E-mail: [zmzou@tmmu.edu.cn](mailto:zmzou@tmmu.edu.cn).

**1. Materials and Methods**

**1.1 Antibodies and reagents**

The GFP-LC3B plasmid was kindly provided by Dr. Tamotsu Yoshimori (Department of Cell Biology, National Institute for Basic Biology, Precursory Research for Embryonic Science and Technology, Okazaki, Japan). Roswell Park Memorial Institute (RPMI) 1640 Medium (SH30809.01B) and fetal bovine serum ([SH30370.03](http://www.bioon.com.cn/reagent/show_product.asp?id=1237790)) were purchased from Hyclone Laboratories (South Logan, UT, USA). A Cell Counting Kit (CCK-8; CK04) was purchased from Dojindo Laboratories (Kumamoto, Japan). 3-MA (M9281), CC (P5499), and antibody against LC3B (L7543) were purchased from Sigma-Aldrich (St. Louis, MO, USA). PI (ST512), Fluo-4 AM (S1060), RAPA (S1842), DCFH-DA (S0033S), phosphate-buffered saline (PBS, C0221A), antibodies against MMP9 (AF5234) and CTSD (AF1645) were purchased from the Beyotime Institute of Biotechnology (Haimen, China). CQ (HY-17589), CPZ (HY-15640), SBI (HY-16966), STO-609 (HY-19805), BafA1 (HY-100558), and digoxin ([HY-B1049](https://www.medchemexpress.com/Digoxin.html)) were purchased from Med Chem Express (Monmouth Junction, NJ, USA). Mechlorethamine hydrochloride (a kind of NM, K900001X) was obtained from Dibo (Shanghai, China). Antibodies against TRPV1 (sc-12503), CaMKKβ (sc-517319) and COX2 (sc-376861) were obtained from Santa Cruz Biotechnology, whereas antibodies against pmTOR (2974), mTOR (4517), pULK1 (14202), pCaMKKβ (12818) and ATG5 (12994) were obtained from Cell Signaling Technology, Inc (Beverly, MA, USA). Antibodies against LAMP1 (ab24170), AMPK (ab32047), pAMPK (ab-133448), TRPV1(ab31895), ULK1 (ab128859), and p62 (ab56416/ ab109012)) were purchased from Abcam (Cambridge, UK) and antibody against ACTB/β-actin (TA-09) was obtained from Zhongshan Jinqiao Biotechnology Co (Beijing, China). LTG (L7526), LysoSensor Green DND-189 (L7535), Premo™ Autophagy Tandem Sensor RFP-GFP-LC3B (P36239), and Lipofectamine™ 2000 transfection reagent (11668-019) were purchased from Invitrogen (Carlsbad, CA, USA).

**1.2 Cell culture**

HaCaT cells, a human skin epithelial cell line, were purchased from the cell bank of the Institute of Cell Biology, Chinese Academy of Science (Shanghai, China) and cultured in RPMI 1640 medium supplemented with 10% fetal bovine serum at 37°C in a humidiﬁed atmosphere containing 5% CO_2_. All experiments were performed following 3–6 passages when the cells reached ~80–90% confluence.

**1.3 Animals and treatments**

About 6-week-old male SKH1 hairless mice, weighing 24–26 g, were purchased from the Shanghai Public Health Clinical Center (Shanghai, China) and maintained at a controlled temperature (22 ± 2°C) and a 12-h light/dark period, with *ad libitum* access to water and food.

**1.4 Cell viability measurement**

The CCK-8 detection kit was used to measure cell viability as previously described.[^1^](#_ENREF_1) Briefly, HaCaT cells were seeded in a 96-well microplate (Corning Life Sciences; 3650) at a density of 5,000 cells/well and then treated with NM for 24 h at a series of concentrations (0.1, 1, 5, 10, 20, 50, 100, and 200 μM). Subsequently, CCK-8 solution (20 μL/well) was added to the wells and the plate was incubated at 37°C for 1.5 h. Viable cells were counted by absorbance measurements with a monochromator microplate reader (Safire II; Tecan Group Ltd., Männedorf, Switzerland) at a wavelength of 450 nm. The optical density value at 450 nm was reported as the percentage of cell viability in relation to the control group (set as 100%).

**1.5 Cell death assay**

The cells were trypsinized with 0.5 ml of 0.25% trypsin for 3 min, collected, and resuspended in 1 ml of PBS. The cells were then incubated with 0.5 ml staining solution (10 mg/mL PI) at 37ºC for 30 min in the dark. Cell death was measured by fluorescence microscope (Olympus IX-71) or ﬂow cytometry (BD FAC Scan Flow cytometer)

**1.6 Western blot analysis**

Cells and skin tissues were collected, lysed and subjected to western blotting as described previously. [^1^](#_ENREF_1) Briefly, 40~100 µg of protein was resolved by 10-15% SDS-PAGE and then electroblotted onto polyvinylidene difluoride membranes for western blot analysis. Blots were probed with 1:1,000-diluted primary antibodies overnight at 4ºC, followed by horseradish peroxidase-conjugated secondary antibodies ([Thermo Scientific Lab Vision;](http://www.thermoscientific.com/ecomm/servlet/search?searchType=0&searchSubType=6&N=4294967135 4294967089&Ne=4294967089&keyWord=rabbit+secondary+antibodies) 31340 and 31455). Protein bands were visualized using the enhanced chemiluminescence system and densitometric analysis was performed using Scion Image-Release Beta 4.02 software (<http://scion-corporation.software.informer.com/>).

**1.7 RFP-GFP-LC3 assay**

Premo™ Autophagy Tandem Sensor RFP-GFP-LC3B, which is a lentivirus carrying expression cassettes that encode tandem fluorescence-tagged LC3B, was used to evaluate the number of autophagosomes and autolysosomes following the manufacturer’s instructions. Briefly, 1 × 10^5^ HaCaT cells were grown on glass-bottom dishes and infected with lentivirus for 24 h. Then, the HaCaT cells were treated with BafA1 (10 nM) for 1 h following the addition of NM (20 μM) for another 24 h. All samples were examined under a ZEISS LSM 780 confocal laser scanning microscope (ZEISS, Germany) equipped with a 40 × oil immersion objective.

**1.8 LTG, LysoSensor Green DND-189 and DCFH-DA staining**

HaCaT cells were cultured overnight on glass-bottom dishes at a density of 10,000 cells/dish or in 96-well microplate at a density of 5,000 cells/well and then exposed to the various indicated treatments. Thereafter, cells were washed twice with fresh medium and loaded with LTG (50 nM), LysoSensor Green DND-189 (1 μM) or DCFH-DA (10 μM) for 10~15 min in humidified air at 37˚C in RPMI 1640 culture medium without fetal bovine serum, respectively. Afterward, the cells were washed three times with PBS and fluorescence intensity was measured by a ZEISS LSM 780 confocal laser scanning microscope (ZEISS, Germany) or was quantified using an Infinite™ M200 Microplate Reader (Tecan Group Ltd.).

**1.9 Measurement of GFP-LC3B dots**

HaCaT cells were transfected with plasmids expressing GFP-LC3B. After 24 h, cells were exposed to various indicated treatments. Then cells were washed with PBS, fixed by incubation for 20 min at 37°C in 4% paraformaldehyde, permeabilized with 0.1% (v/v) Triton X-100, and washed with PBS containing 2% fetal bovine serum albumin. All steps were performed at room temperature. A Radiance 2000 laser scanning confocal microscope (Bio-Rad, Hercules, CA) was used for confocal microscopy analysis.

**1.10 Transmission electron microscopy**

HaCaT cells were collected and fixed in 2% paraformaldehyde and 0.1% glutaraldehyde in 0.1 M sodium cacodylate for 2 h, post-fixed with 1% OsO4 for 1.5 h, washed, and stained for 1 h in 3% aqueous uranyl acetate. The samples were then washed again, dehydrated with graded alcohol, and embedded in Epon-Araldite resin (Canemco & Marivac, 034). Ultrathin sections were cut on a ultramicrotome (Reichert-Jung, Inc., Cambridge, UK), counterstained with 0.3% lead citrate, and examined on a transmission electron microscope (model no.: EM420; Koninklijke Philips Electronics N.V., Amsterdam, The Netherlands).

**1.11 siRNA assay**

siRNAs for *ATG5* (human, sc-41445), *AMPK* (human, sc-45312), *CaMKK-β* (human, sc-38955) and *TRPV1* (human, sc-36826) were purchased from Santa Cruz Biotechnology along with control siRNA (sc-44230). Lipofectamine 2000 was diluted in Opti-MEM® I reduced serum medium (31985070, Gibco) according to the manufacturer’s protocol. HaCaT cells were transfected with 100 nM siRNA with 4 μg plasmid for 5-7 h according to the manufacturer’s protocol. Then, the cells were switched into RPMI 1640 medium and incubated for an additional 24 h. Where indicated, cells were treated with NM (20 µM) for 24 h. Thereafter, cells were harvested and western blot analysis was performed.

**1.12 Detection of intracellular Ca^2+^ content**

The intracellular Ca^2+^ content was determined using Fluo-4 AM, according to the manufacturer’s instructions. HaCaT cells were cultured in 96-well microplates or cover glasses and treated as indicated. Next, cells were loaded with Fluo-4 AM (2 μM) at 37ºC for 30 min in the dark and washed gently three times with warm phosphate buffer saline. Fluorescence intensity of intracullar Ca^2+^ was measured by an Infinite^TM^ M200 Microplate Reader (Tecan Group Ltd.) or imaging with a Radiance 2000 laser scanning confocal microscope (Bio-Rad, Hercules, CA).

**1.13 Detection of** **cellular ATP levels**

A luciferase-based enhanced ATP assay kit (S0027, Beyotime) was used to determine the ATP levels in cells according to the manufacturer’s instructions. HaCaT cells were cultured in 6-well plates and treated as indicated. Next, cells were washed with cold PBS and lysed immediately in 100 μL lysis buffer on ice. The lysate was collected and centrifuged at 12,000 g for 5 min. In a 96-well plate, 20 μL of each supernatant was added into the wells containing 100 μL ATP detection working dilution. The luminescence was detected by a multifunctional microplate reader (SpectraMax i3x, Molecular Devices, USA), and the protein concentration of each group was also determined and used to calibrate the ATP levels in cells. Finally, the cellular ATP levels were presented as percentage of control.

**1.14 H&E staining and histopathological analysis**

At 1 or 3 d after NM exposure, the skin wounds and nearby tissues were cut off and fixed in 4% paraformaldehyde containing 0.1% DEPC for histology analysis. The subsequent preparation for paraffin block was taken as routine program. Slice thickness was limited in 5 μm for H&E staining and microscopically (Zeiss microscope, Germany) evaluated for histopathological features such as epidermal thickness, parakeratosis, epidermal denuding, epidermal death and micriovesication as described previously. [^2^](#_ENREF_2)

**1.15 Immunohistological analysis**

At 1 d after NM exposure, the wounded skin and nearby control tissues were excised in full-thickness and ﬁxed in 4% paraformaldehyde. The paraﬃn blocks of tissue were subsequently prepared. For immunohistological analysis, the slice thickness was 2-3 μm for tissue observation and was incubated with rabbit anti-LC3B antibody (1:400) overnight at 4°C. Target antigens were displayed using a commercial kit (AR1022, **Boster** Biotechnology Co, Wuhan, China) based on streptavidin–biotin complex. The brown color stained by DAB indicated a positive signal, and the blue color stained by hematoxylin indicated the nucleus.

**1.16 Immunofluorescence analysis**

At 1 d after NM exposure, the wounded skin and nearby control tissues were excised in full-thickness and embedded in OCT at -20°C. Then, the tissues were sectioned at a thickness of 6-8 μm. For immunofluorescence analysis, the sections were washed three times with PBS for 5 min, permeabilized with 0.3% (v/v) Triton X-100 for 10 min and blocked in 3% BSA for 1 h at room temperature. Thereafter, the sections were incubated with rabbit anti-LC3B antibody (1:200) overnight at 4°C. Then, the sections were incubated with the appropriate Alexa Fluor® 488 goat anti-rabbit IgG (H+L) antibody (A11034, Invitrogen) for 2 h at room temperature. DAPI staining solution (C1005, Beyotime) was used to staining the nuclei for 10 min at room temperature. Finally, the sections were mounted on glass slides, and digital images were acquired at 100 × magnification using a ZEISS LSM800 confocal laser scanning microscope (ZEISS, Germany).


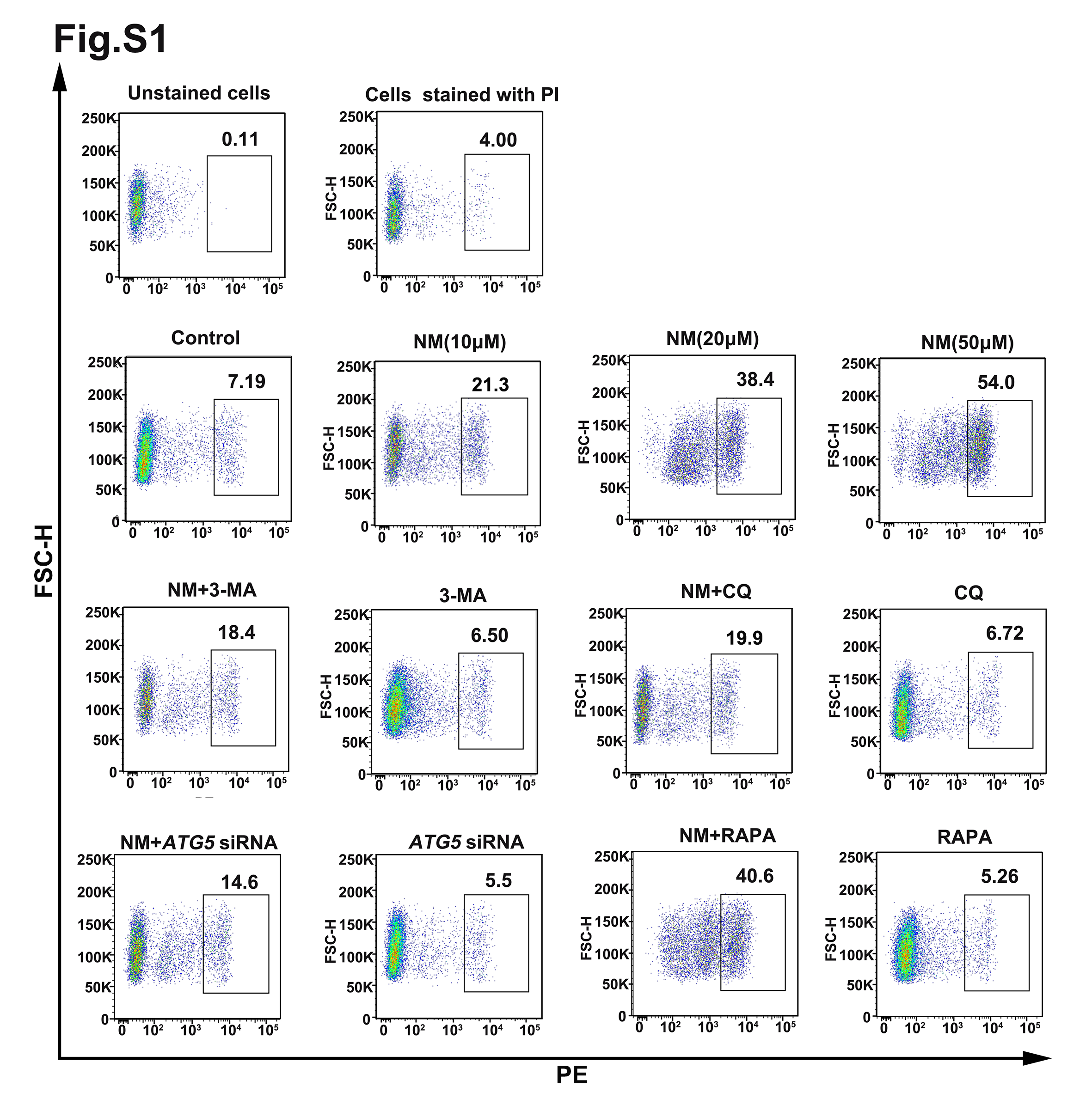


**Figure S1. Cell death measured by ﬂow cytometry, Related to Fig.1, Fig.3 and Fig.5.** Cells were treated with different concentrations (10, 20 and 50 μM)) of NM for 24 h. Meanwhile, cells were also treated with 3-MA (5 mM), CQ (5 µM), or RAPA (20 nM) for 1 h following the addition of NM (20 µM) for another 24 h. Moreover, ATG5 was knocked down by *ATG5* siRNA as described in the Materials and Methods. At 24 h post-transfection, cells were treated with NM (20 µM) for 24 h. Cells were collected and cell death was determined with PI staining by ﬂow cytometry as described in the Materials and Methods section.


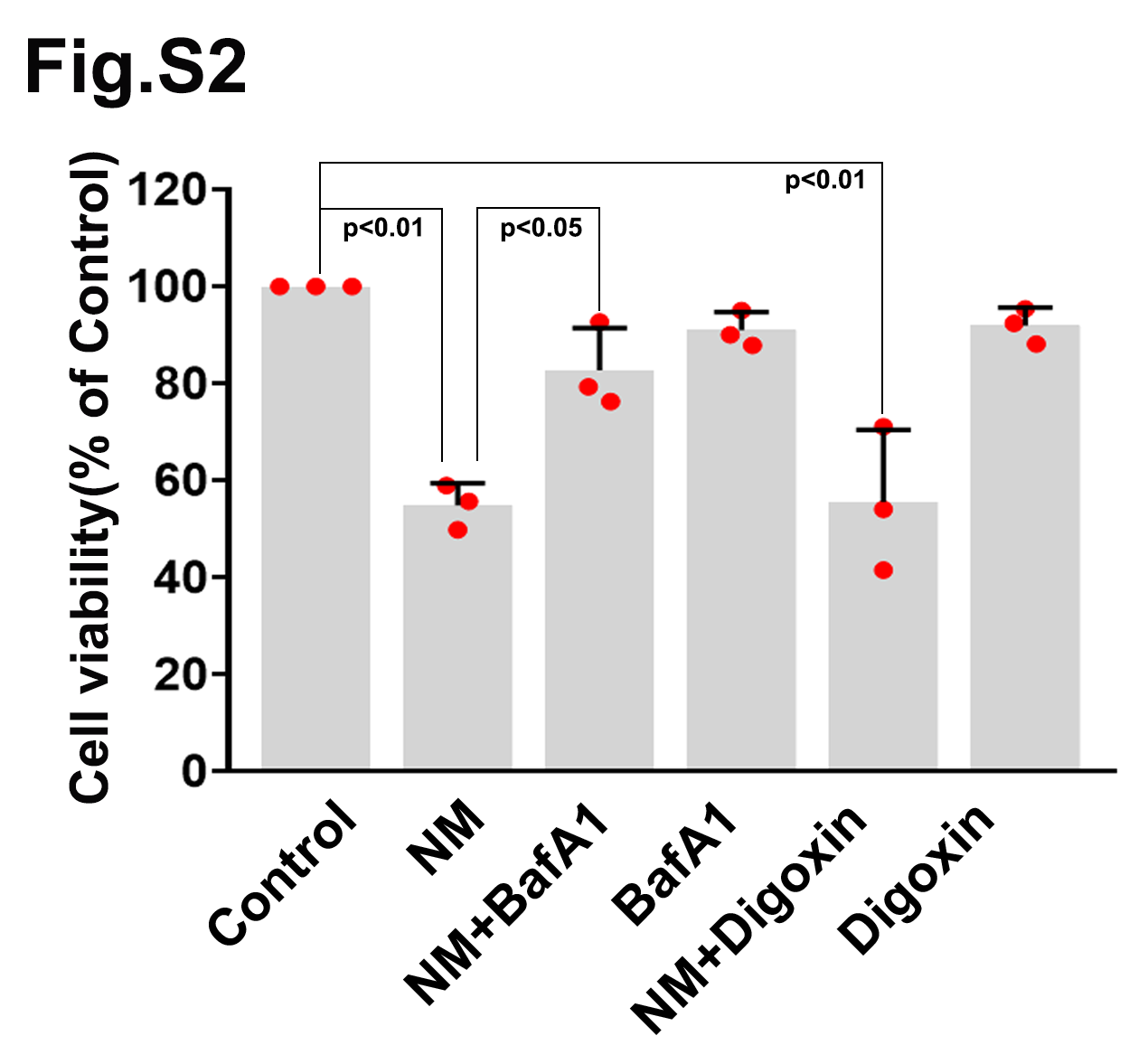


**Figure S2. The effect of BafA1 and digoxin on NM-induced keratinocyte cell death, Related to Fig.3.**

Cells were treated with BafA1 (10 nM) or digoxin (0.1 µM) for 1 h following the addition of NM (20 µM) for another 24 h. Cell viability was measured by a CCK-8 kit as described in the Materials and Methods section. Values are expressed as the means ± SD (n = 3).


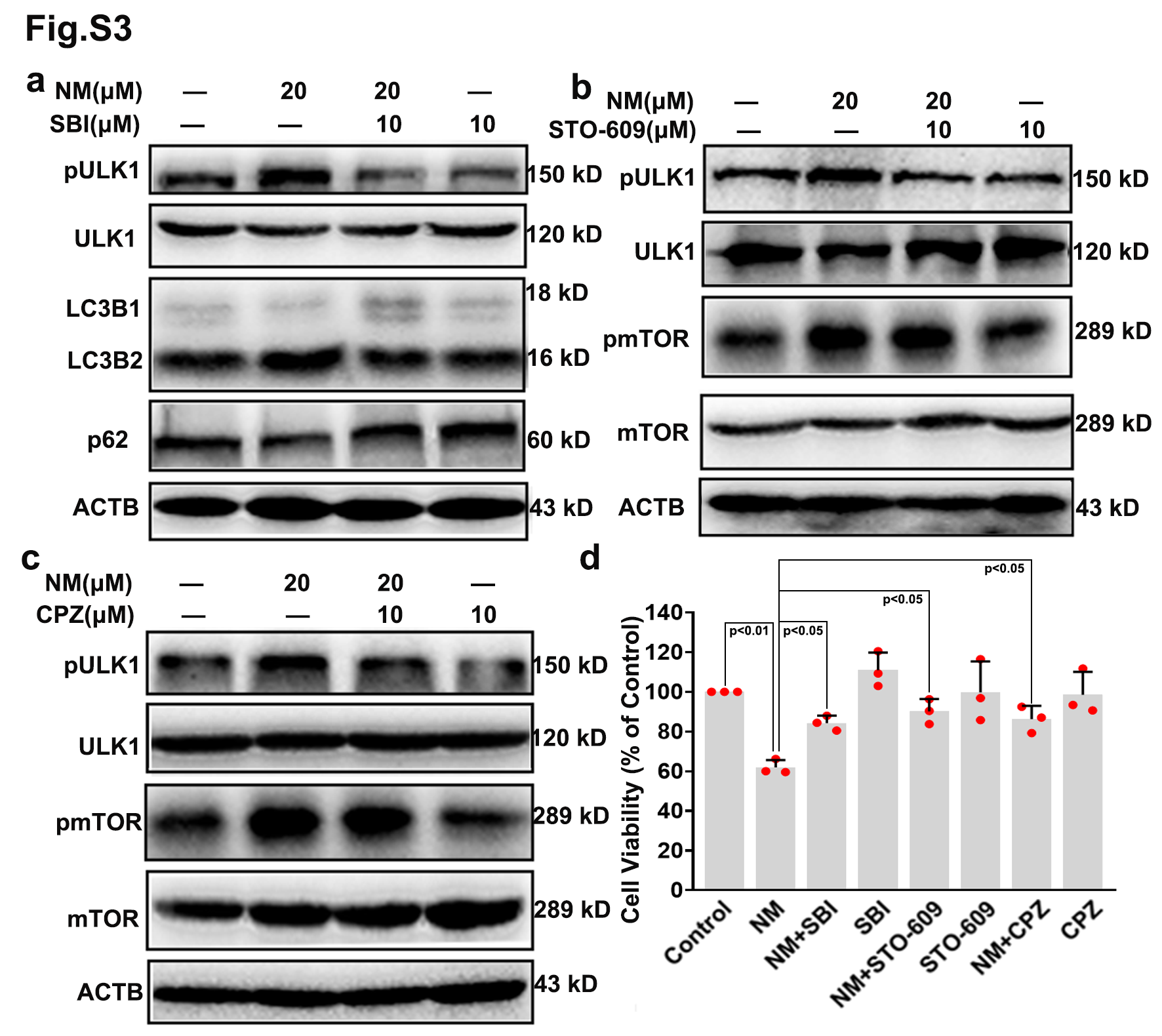


**Figure S3. The effect of SBI, STO-609 and CPZ on NM-induced autophagy, ULK1 and mTOR activation and cell death, Related to Fig.5, Fig.6 and Fig.7.**

Cells were treated with SBI (10 µM), STO-609 (10 µM), or CPZ (10 µM) for 1 h following the addition of NM (20 µM) for another 24 h. **(a), (b)** and **(c)** Total cell lysates were collected and the levels of indicated proteins were detected by western blotting. (**d**) Cell viability was measured by a CCK-8 kit as described in the Materials and Methods section. Values are expressed as the means ± SD (n = 3).


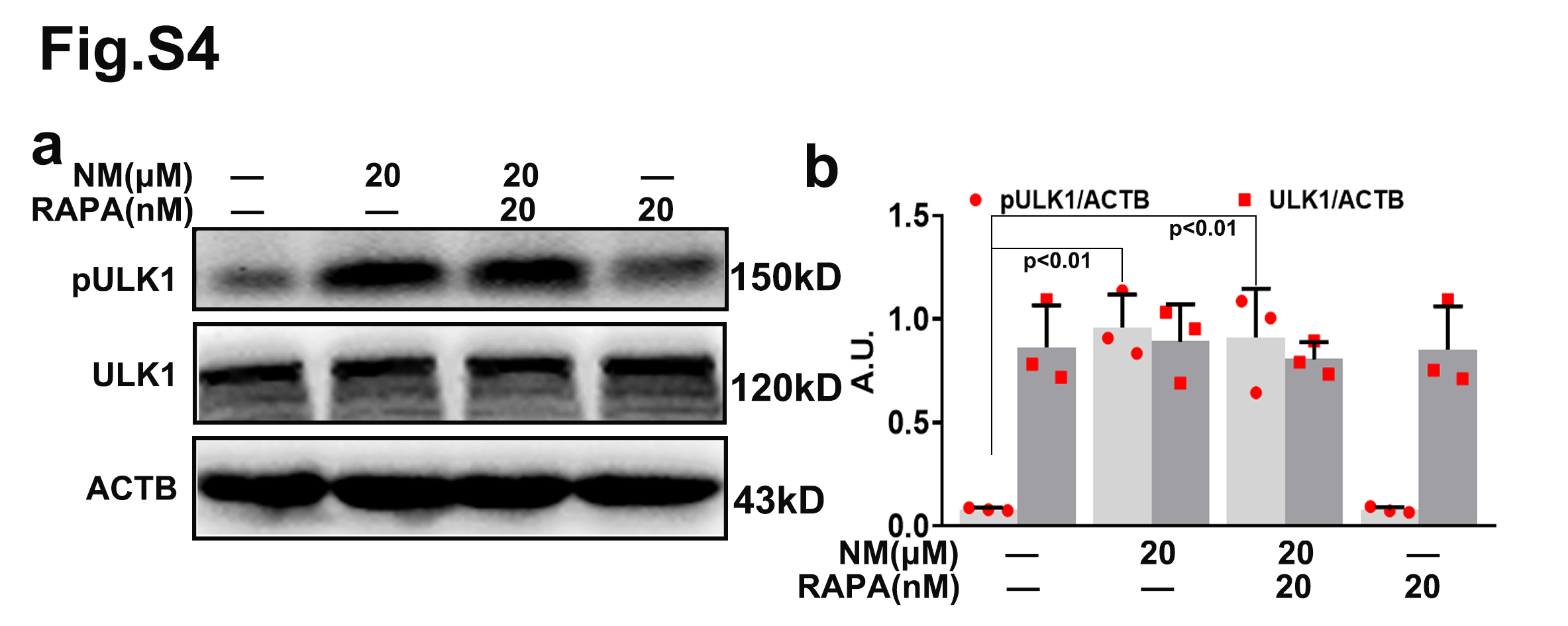


**Figure S4. The effect of RAPA on NM-induced ULK1 activation, Related to Fig.5.** Cells were treated with RAPA (20 nM) for 1 h, following the addition of NM (20 µM) for another 24 h. **(a)** Total cell lysates were collected and the levels of pULK1 and UKL1 were detected by western blotting. **(b)** The bar graphs show the quantification of pULK1 and UKL1. Values are expressed as the means ± SD (n = 3).


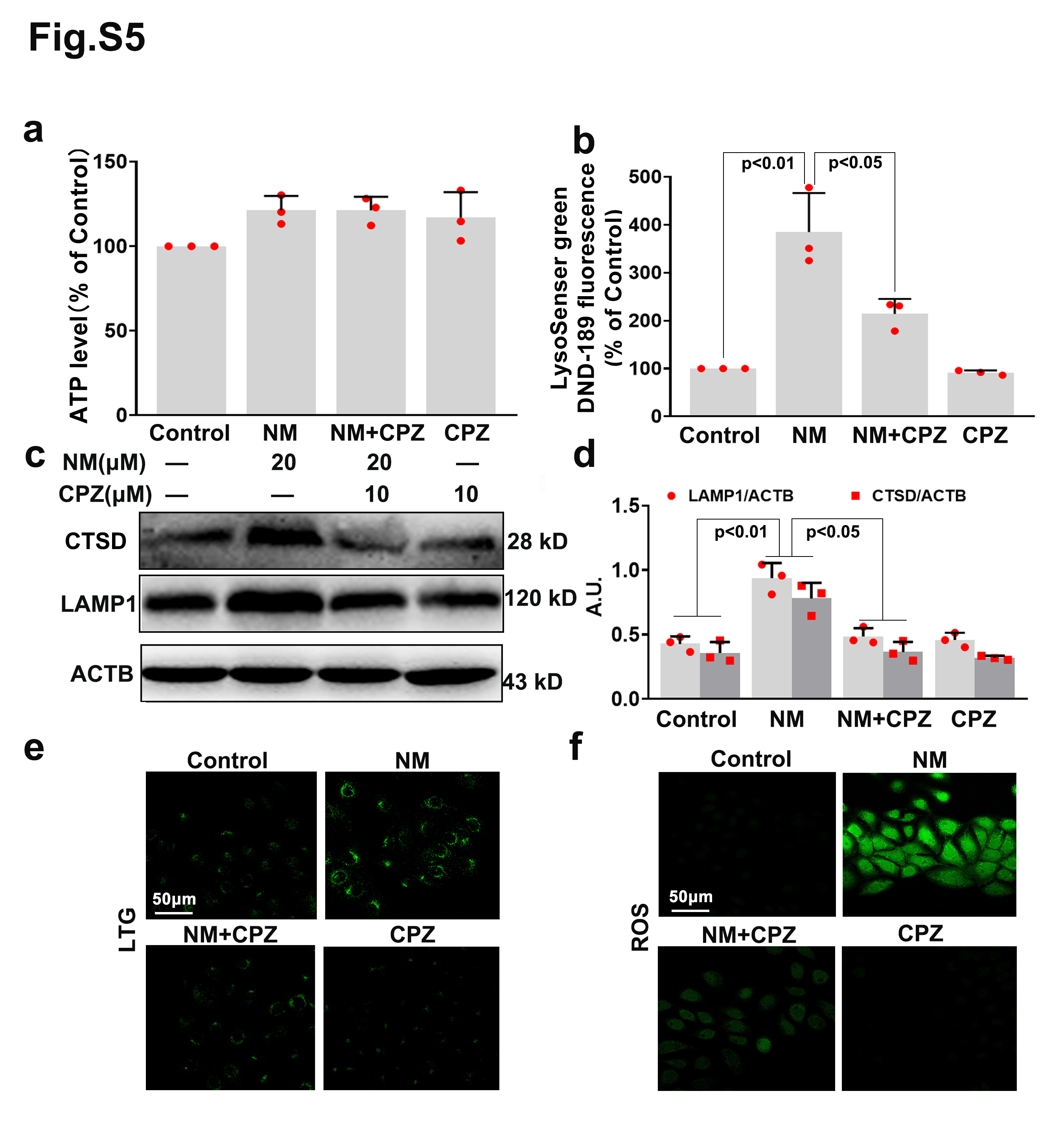


**Figure S5. The effect of CPZ on NM-induced ATP release, lysosomal functions, and ROS generation, Related to Fig.7.** Cells were treated with CPZ (10 μM) for 1 h, following the addition of NM (20 µM) for another 24 h. **(a)** The cellular ATP levels were measured by a luciferase-based enhanced ATP assay kit according to the manufacturer’s instructions as described in the Materials and Methods section. **(b)** LysoSensor DND-189 fluorescence intensity was quantified using an Infinite™ M200 Microplate Reader (Tecan Group Ltd.). **(c)** The expression of LAMP1, CTSD and ACTB were detected by western blotting. (**d**) The bar graph shows the quantification of the indicated proteins. **(e)** LTG and **(f)** DCFH-DA fluorescence intensity was measured by a ZEISS LSM 780 confocal laser scanning microscope (ZEISS, Germany). Values are expressed as the means ± SD (n = 3).


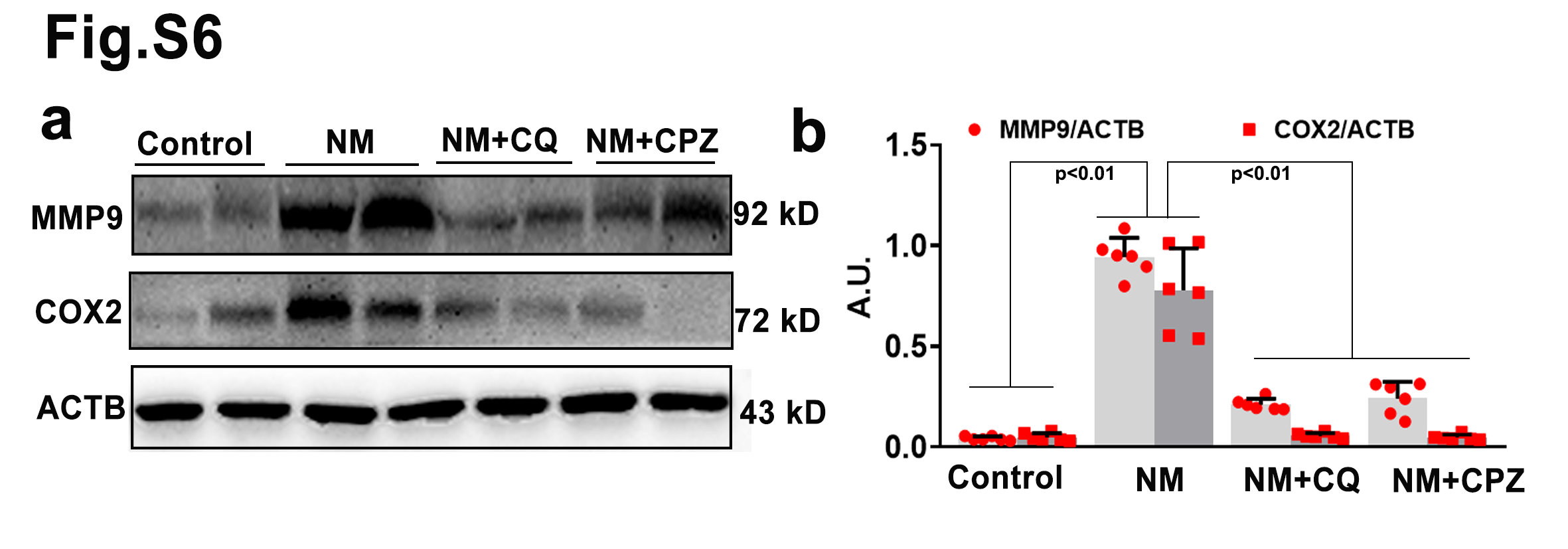


**Figure S6. The effect of CQ and CPZ on NM-induced COX2 and MMP9 expression in skin, Related to Fig.8.** The dorsal skins of 6-week-old male SKH-1 hairless mice with 24 g~26 g weight (n = 6 per group) were treated with CQ (60 mg/kg, i.p.) or CPZ (5 mg/kg, i.p.), then were exposed to 3.2 mg NM in 200 μL acetone as described in the Materials and Methods. The skin tissues were cut off at 72 h post NM exposure and lysed, then western blot analysis was performed. **(a)** The expression of COX2 and MMP9 was measured by western blotting. **(b)** The bar graphs show the quantification of COX2 and MMP9. Values are expressed as the means ± SD (n = 6).

**References**

1 Chen, M. L. *et al.* Resveratrol attenuates vascular endothelial inflammation by inducing autophagy through the cAMP signaling pathway. *Autophagy* **9**, 2033-2045, doi:10.4161/auto.26336 (2013).

2 Jain, A. K. *et al.* Flavanone silibinin treatment attenuates nitrogen mustard-induced toxic effects in mouse skin. *Toxicol Appl Pharmacol* **285**, 71-78, doi:10.1016/j.taap.2015.03.009 (2015).
